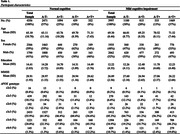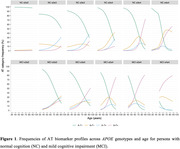# Prevalence of cerebrospinal fluid AT biomarker profiles according to APOE genotype and across age in persons without dementia

**DOI:** 10.1002/alz.091306

**Published:** 2025-01-09

**Authors:** Jolien van der Velden, Stephanie J. B. Vos, Betty M. Tijms, Julie Elisabeth Oomens, Nancy N Maserejian, Pieter Jelle Visser, Willemijn J. Jansen, Amyloid Biomarker Study Group

**Affiliations:** ^1^ Alzheimer Center Limburg, School for Mental Health and Neuroscience, Maastricht University, Maastricht Netherlands; ^2^ Alzheimer Center Amsterdam, Department of Neurology, Amsterdam Neuroscience, Vrije Universiteit Amsterdam, Amsterdam UMC, Amsterdam Netherlands; ^3^ Biogen, Cambridge, MA USA; ^4^ Alzheimer Center and Department of Neurology, Amsterdam Neuroscience Campus, VU University Medical Center, Amsterdam Netherlands

## Abstract

**Background:**

Amyloid‐b deposition and tau tangle formation are the key pathologies of Alzheimer’s disease (AD). The presence of these pathologies in cerebrospinal fluid (CSF) biomarkers is used for a biological diagnosis of AD. It remains unclear how the prevalence of AT biomarker profiles depends on apolipoprotein E (APOE) genotype. A better understanding of the AT biomarker profiles across APOE genotypes and age in the pre‐dementia stages will help inform strategies to prevent or delay AD progression in those with different APOE genotypes.

**Methods:**

Participants with normal cognition (NC; n = 4356) or mild cognitive impairment (MCI; n = 3997) were selected from 45 studies that participated in the Amyloid Biomarker Study based on availability of data on age, CSF amyloid‐b_42_ (A), CSF phosphorylated tau 181 (T), and APOE genotype (e2e2, e2e3, e2e4, e3e3, e3e4, e4e4). Based on (ab)normal values for A (data‐driven) and T (center‐specific cutoffs) participants were placed into one of four AT biomarker profiles: A‐T‐, A+T‐, A‐T+, or A+T+. A Markov‐Chain‐Monte‐Carlo generalized linear mixed model was performed separately for NC and MCI to evaluate the association of AT profile frequency with age and APOE genotype. Center was included as random effect.

**Results:**

The NC and MCI groups had an average age of 65.1 years (SD = 10.8; 59% female) and 69.3 years (SD = 8.4; 48% female) respectively. Most participants had APOE e3e3 genotype (NC 56%, MCI 45%; Table 1). In both groups, the association of AT profiles with APOE genotype changed across age (both p < .001; Figure 1), except for participants with NC and APOE e2e2 genotype. For NC with APOE e2e3 and e3e3 genotypes, A‐T+ was more frequent in older age than A+T‐ or A+T+. In APOE e3e4 and e4e4 genotypes A+T‐ frequency plateaued around 65 years of age, while A+T+ frequency strongly increased. For MCI, A+T+ was most frequent with aging across APOE e2e4, e3e3, e3e4, and e4e4 genotypes.

**Conclusion:**

Frequencies of AT biomarker profiles changed substantially across APOE genotypes and age in NC and MCI. Studies recruiting participants based on AT biomarker profiles should take age and APOE genotype into account.